# Highly Efficient Iron Oxide Nanoparticles Immobilized on Cellulose Nanofibril Aerogels for Arsenic Removal from Water

**DOI:** 10.3390/nano11112818

**Published:** 2021-10-23

**Authors:** Md Musfiqur Rahman, Islam Hafez, Mehdi Tajvidi, Aria Amirbahman

**Affiliations:** 1 Laboratory of Renewable Nanomaterials, School of Forest Resources, University of Maine, 5755 Nutting Hall, Orono, ME 04469, USA; md.musfiqur.rahman@maine.edu (M.M.R.); mehdi.tajvidi@maine.edu (M.T.); 2 Department of Civil, Environmental and Sustainable Engineering, Santa Clara University, 500 El Camino Real, Santa Clara, CA 95053, USA; aamirbahman@scu.edu

**Keywords:** cellulose nanofibrils, iron oxide nanoparticles, aerogel, arsenic, water treatment

## Abstract

The application and optimal operation of nanoparticle adsorbents in fixed-bed columns or industrial-scale water treatment applications are limited. This limitation is generally due to the tendency of nanoparticles to aggregate, the use of non-sustainable and inefficient polymeric resins as supporting materials in fixed-bed columns, or low adsorption capacity. In this study, magnesium-doped amorphous iron oxide nanoparticles (IONPs) were synthesized and immobilized on the surface of cellulose nanofibrils (CNFs) within a lightweight porous aerogel for arsenic removal from water. The IONPs had a specific surface area of 165 m^2^ g^−1^. The IONP-containing CNF aerogels were stable in water and under constant agitation due to the induced crosslinking using an epichlorohydrin crosslinker. The adsorption kinetics showed that both As(III) and As(V) adsorption followed a pseudo second-order kinetic model, and the equilibrium adsorption isotherm was best fitted using the Langmuir model. The maximum adsorption capacities of CNF-IONP aerogel for As(III) and As(V) were 48 and 91 mg As g-IONP^−1^, respectively. The optimum IONP concentration in the aerogel was 12.5 wt.%, which resulted in a maximum arsenic removal, minimal mass loss, and negligible leaching of iron into water.

## 1. Introduction

Arsenic is one of the most toxic elements that can be found in groundwater at high concentrations in many locations across the globe [1]. Long-term exposure to arsenic from drinking water is associated with health issues such as cancers, skin lesions, and cardiovascular diseases [2]. Arsenic is a metalloid that has two major oxidation states in an ambient environment: arsenite, As(III), and arsenate, As(V). According to the United States Environmental Protection Agency (US-EPA), the maximum contaminant level (MCL) for arsenic in drinking water is 10 μg L^−1^.

Arsenic can be removed from water by techniques such as reverse osmosis, oxidation, ion exchange, and coagulation–flocculation [3]. These technologies are efficient but are often costly and are based on non-sustainable materials. Adsorption-based techniques have the potential to effectively treat drinking water if an appropriate and efficient adsorbent is developed [4]. A number of studies have focused on the development of cost-effective and sustainable adsorbents for arsenic removal [5]. Iron oxide-based nanoparticles typically have a high affinity towards arsenic. Crystalline iron oxide nanoparticles such as hematite (α-Fe_2_O_3_), magnetite (Fe_3_O_4_), or maghemite (γ-Fe_2_O_3_) have relatively low specific surface areas (<100 m^2^ g^−1^) [4,6]. Amorphous iron oxide nanoparticles (IONPs) tend to have a higher surface area than crystalline nanoparticles; however, their transition to other crystalline phases over time and temperature limits their application [7,8,9]. Doping IONPs with another metal ion could inhibit their phase transition and retain their amorphous nature, hence maintaining the increased surface area [9,10,11]. Another common challenge with using IONPs is their tendency to aggregate, which decreases their specific surface area [12]. This means that the application of nanoparticle adsorbents in fixed-bed columns or at an industrial scale is limited. Researchers have attempted to utilize nanoparticulate aggregates in fixed-bed columns [13,14,15]. In these attempts, the adsorption capacity was often sacrificed because of the increased extent of aggregation.

Cellulose nanomaterials have emerged as promising materials in environmental applications. Cellulose is a linear polysaccharide composed of glucose units connected through β 1–4 glycosidic bonds [16]. Cellulose nanofibrils (CNFs) are generally produced by mechanical refining or other mechanical size reduction methods such as microfluidization, homogenization, or grinding [17]. The diameter of the fibrils is typically 5–50 nm, and the fibrils can be several μm in length [16].

A large number of scientific reports have been published to prove the concept of nanocellulose-based materials for a variety of water and wastewater treatment processes, including the removal of toxic heavy metals and dyes [18]. The ongoing research on nanocellulose-based materials for sustainable water treatment has largely focused on the development of chemically functionalized materials such as TEMPO (2,2,6,6-tetramethylpiperidine-1-oxyl)-oxidized, carboxylated, sulfonated, phosphorylated, and amino-functionalized nanocellulose to enhance the adsorption capacity [19]. The functionalization route remains a challenging task due to the complexity of the process and the low adsorption capacity.

Immobilizing the IONPs on the surface of cellulose fibrils could be a promising solution to tackle the two challenges mentioned above: aggregation of nanoparticles and their limited application in fixed-bed columns. CNFs have the potential to decrease the extent of IONP aggregation owing to their stable aqueous suspension and nanoscale dimensions. In such a case, drying a stable suspension of CNFs and IONPs into a foam or aerogel structure could result in uniformly distributed IONPs within the CNF network with minimal aggregation. A few attempts have been made to incorporate iron oxide nanoparticles into porous CNF structures [20,21]. Although the incorporation of the nano-entities seemed feasible, several issues have been identified. First, the studies lacked adequate characterization of adsorbent stability under wet conditions, which is essential given their intended application in water treatment. Second, information relative to the stability of nanoparticles within the cellulose structures remains to be elucidated. Third, a favorable adsorption capacity was obtained by incorporating a high load of iron oxide nanoparticles within the aerogel (36%) without investigating the structural stability of the adsorbents in water [20].

In the present work, a novel protocol to produce porous CNF structures loaded with uniformly distributed IONPs with minimal aggregation and considerably high specific surface area was studied. The IONPs were immobilized on CNFs, and the adsorbents were tested for their adsorption performance towards As(III) and As(V). In addition, extensive physical and chemical characterizations of the CNF-IONP aerogels were conducted, as well as an assessment of their stability under various IONP loadings.

## 2. Materials and Methods

### 2.1. Materials

CNFs were provided by the Process Development Center (PDC) of the University of Maine (Orono, ME, USA). The 3 wt.% CNFs were produced by mechanically refining bleached softwood Kraft pulp. This is the standard grade of CNFs produced by the PDC and contains 90% fines. In this context, the fines content indicates the percentage of fibers with lengths < 200 µm. Polycup^TM^ (polyamide-epichlorohydrin) 5150 crosslinker (26 wt.%) was supplied by Solenis (Wilmington, DE, USA). Anhydrous ferric chloride (FeCl_3_; 98%) and magnesium chloride (MgCl_2_; 99%) were purchased from Alfa Aeser (Haverhill, MA, USA). Anhydrous ethyl alcohol (C_2_H_5_OH; 99.5%) was obtained from Acros Organics (Jair Lawn, NJ, USA). HEPES (4-(2-hydroxyethyl)-1-piperazineethanesulfonic acid) buffering agent (≥99.5%), sodium chloride (NaCl; ≥99%), sodium hydroxide (NaOH; ≥97%), hydrochloric acid (HCl; 37%), sodium (meta) arsenite (NaAsO_2_), and sodium arsenate dibasic heptahydrate (Na_2_HAsO_4_.7H_2_O; ≥98%) were purchased from Sigma-Aldrich (St. Louis, MO, USA). All chemicals and solvents were used without any further purification.

### 2.2. Synthesis of Mg-Doped Amorphous IONPs

To synthesize the Mg-doped amorphous IONPs, 0.09 M FeCl_3_ and 0.01 M MgCl_2_ solutions were prepared by dissolving 1.02 g FeCl_3_ and 0.066 g MgCl_2_ salts together in 70 mL of ethanol [22]. A 20 mL 2.3 M ethanolic NaOH solution was prepared by dissolving 1.84 g solid NaOH. Ten mL of 2.3 M ethanolic NaOH solution was added to the salt mixture, which resulted in a red yellowish precipitate of the amorphous Mg-doped ferric hydroxide, (Fe, Mg)_x_(OH)_y_. The solution was continuously ultrasonicated by a Branson 450 sonicator (Branson Ultrasonics Corporation; Radnor, PA, USA) for 1 h to break up the aggregates. The reaction mixture was then transferred to a 100 mL Teflon-lined autoclave container and placed in a preheated oven at 150 °C for 2 h. During the heating process, simultaneous nucleation and homogeneous heating further decreased the particle size [9]. Subsequently, the autoclave was cooled to room temperature. The IONP suspension was washed with distilled water 2–3 times using a centrifuge to remove ethanol and other dissolved components until pH 7.0 was reached. Finally, the volume of the IONP suspension was adjusted to 50 mL by adding distilled water, and the suspension was stored at 2–5 °C.

### 2.3. Preparation of CNF-IONP Adsorbent

Initially, a batch of 1 wt.% CNF suspension was mixed with Polycup^TM^ crosslinker (5 wt.% of the total dry mass of CNFs) and stirred with a magnetic stirrer at 300 rpm for 5 min to ensure a homogeneous distribution of the crosslinker. CNF aerogels containing IONP content of 12.5 wt.% were prepared by mixing IONP and CNF suspensions according to the amounts listed in Table S1. The suspension mixture was mechanically stirred with a magnetic stirrer at 300 rpm followed by continuous ultrasonication for 5 min. Finally, the CNF-IONP suspension mixture was poured into a cylindrical plastic mold (height: 2.5 cm and diameter: 1.3 cm) and freeze-dried using a Harvest Right freeze-dryer (North Salt Lake, UT, USA). The temperature cycles of the freeze dryer were −34.4, −6.7, 4.4, 15.6, and 32.2 °C for 8, 10, 8, 3, and 3 h, respectively. A schematic illustration that describes the synthesis process is presented in Figure S1. After freeze-drying, the CNF-IONP aerogel adsorbents were heated in a vacuum oven (25 mm Hg = 86 kPa) at 105 ± 2 °C to induce crosslinking. The proposed reaction mechanism is presented in Figure S2.

### 2.4. Characterization

A Panalytical X’Pert PRO X-ray diffractometer (Royston, UK) was used to assess the nature of the nanoparticles and the crystalline structure of the CNF aerogels. The XRD anode material was Cu with Kα at a wavelength of 1.54 nm. The generator voltage and current were 40 kV and 40 mA, respectively. The scan step size and 2θ range were 0.05° s^−1^ and 10–80°, respectively. The baseline correction, smoothing, and background subtraction for the XRD data were performed using Origin Pro 2021 software.

The surface morphology of CNF aerogels before and after IONP immobilization was assessed using a Zeiss Nvision 40 scanning electron microscope (SEM; Oberkochen, Germany). The aerogel samples of a thickness of 2–4 mm were prepared by slicing through the middle section using a sharp blade. Prior to imaging, all the samples were sputter-coated by a thin layer of gold–palladium and scanned at an accelerating voltage of 3 kV.

Energy-dispersive X-ray spectroscopy (EDS; iXRF model 550i AMRay 1820, Bedford, MA, USA) was used for the elemental mapping of Fe, Mg, C, and O atoms at the CNF-IONP surface. An accelerating voltage of 20 kV was maintained for the EDS analysis.

The specific surface areas (SSA) of freeze-dried CNF aerogel and IONP samples were measured by the Brunauer, Emmett, and Teller (BET) nitrogen adsorption method using an ASAP 2020 instrument (Micromeritics; Norcross, GA, USA). The CNF aerogel and IONP samples were degassed in a vacuum for 5 h at 75 and 130 °C, respectively.

The ATR-FTIR analysis was performed using a PerkinElmer Spectrum Two™ FTIR spectrophotometer (Shelton, CT, USA) to evaluate the nature of the interaction between the CNFs and IONPs in the aerogel. The data obtained for CNF and CNF-IONP aerogels were normalized with respect to the wavenumber 1055 cm^−1^, which represents the stretching vibration of the cellulose backbone (not altered by the crosslinking reaction).

A Nano ZS90 Zetasizer (Malvern, UK) was used to measure the zeta potentials of CNFs and IONPs in water. The isoelectric points (IEPs) were determined by measuring the zeta potentials at pH 3–11. Relatively low concentrations (~0.1 wt.%) of CNF and IONP suspensions were used with a constant ionic strength of 0.01 M NaCl for all the zeta potential measurements.

The density (*ρ*, g cm^−3^) and porosity (%) of the aerogels were calculated by measuring the void volume (*v*_1_) with an Accupyc II gas pycnometry system (Norcross, GA, USA), the total volume (*v*_2_) using a digital caliper, and corresponding mass (m) according to Equations (1) and (2), respectively.

(1) $$\mathrm{Density}\;\mathrm{of}\;\mathrm{the}\;\mathrm{aerogel},\;\rho =\frac{m}{v_{2}}$$

(2) $$\mathrm{Porosity}\;\left(\%\right)=\left(1-\frac{v_{1}}{v_{2}}\right)\times 100$$

The shape recovery tests were conducted by compressing the aerogels at a pressure of 1.2 kPa using a Dake^®^ manual hydraulic pump. The aerogel was compressed into a 5 mm thin disk and submerged in 80 mL distilled water for 10 s. The aerogel heights before compression (*h_i_*) and after submerging in water (*h_f_*) were recorded. The shape recovery was calculated using Equation (3).

(3) $$\mathrm{Shape}\;\mathrm{recovery}\;\left(\%\right)=\frac{h_{f}}{h_{i}}\times 100$$

The water absorption capacity of the aerogels was determined by calculating the mass differences of aerogels before (*W_i_*) and after (*W_f_*) soaking in 80 mL distilled water for 12 h according to Equation (4).

(4) $$\mathrm{Water}\;\mathrm{absorption}\;\mathrm{capacity}\;\left(g-\mathrm{water}\;g-\mathrm{dry}\;\mathrm{mass}-1\right)=\frac{{(W}_{f}{-W}_{i})}{W_{i}}$$

The mass losses of the CNF and CNF-IONP aerogels were determined gravimetrically after submerging the aerogels into 80 mL distilled water for 12 h with constant agitation and drying in an oven at 75 °C for 5 h. The % of mass loss was calculated by comparing the initial (*m*_1_) and after drying (*m*_2_) dry mass according to Equation (5).

(5) $$\mathrm{Mass}\;\mathrm{loss}\;\left(\%\right)=\left(\frac{m_{1}-m_{2}}{m_{1}}\right)\times 100$$

### 2.5. Arsenic Adsorption Experiments

All batch arsenic adsorption experiments were carried out in 100 mL Falcon tubes filled up to 80 mL at room temperature (~25 °C) under constant agitation (VWR Scientific Products rocking platform model 100, Radnor, PA, USA). For the kinetic experiments, 3 mL of solution was removed at specific time intervals. Samples were acidified immediately after collection by 1% *v*/*v* of concentrated HNO_3_, and the arsenic and iron concentrations were measured by a Thermo Scientific™ Element 2™ ICP-MS (Waltham, MA, USA) calibrated using SLRS-6, a river certified reference material from National Research Council of Canada. The initial arsenic concentration for adsorption kinetic studies was ~300 µg L^−1^ for both As(III) and As(V). For the kinetic experiments involving both As species, the initial IONP concentrations were 16, 31, and 63 mg L^−1^. Samples were collected for analysis at different time intervals up to 12 h. For the equilibrium adsorption experiments, the initial arsenic concentration ranged from 0.055 to 15.89 mg L^−1^ for As(III), and 0.073 to 21.65 mg L^−1^ for As(V). All arsenic solutions were prepared in a 0.01 M HEPES buffer with 0.05 M NaCl. The pH remained constant at 7.0–7.2 throughout all experiments. A set of equilibrium adsorption experiments were conducted at different pH values and at an initial As(V) and As(III) concentration of 1 mg L^−1^ with a dosage of 63 mg of IONP L^−1^. The role of IONP concentration in the CNF-IONP aerogel was investigated by varying the wt.% of IONPs between 1% and 30% and adjusting the CNF concentration accordingly. A concentration of 7.2 mg L^−1^ of As(V) solution was used to perform the analysis with 12 h of contact time under constant agitation.

## 3. Results and Discussion

### 3.1. Arsenic Adsorption Experiments

**Figure 1:**
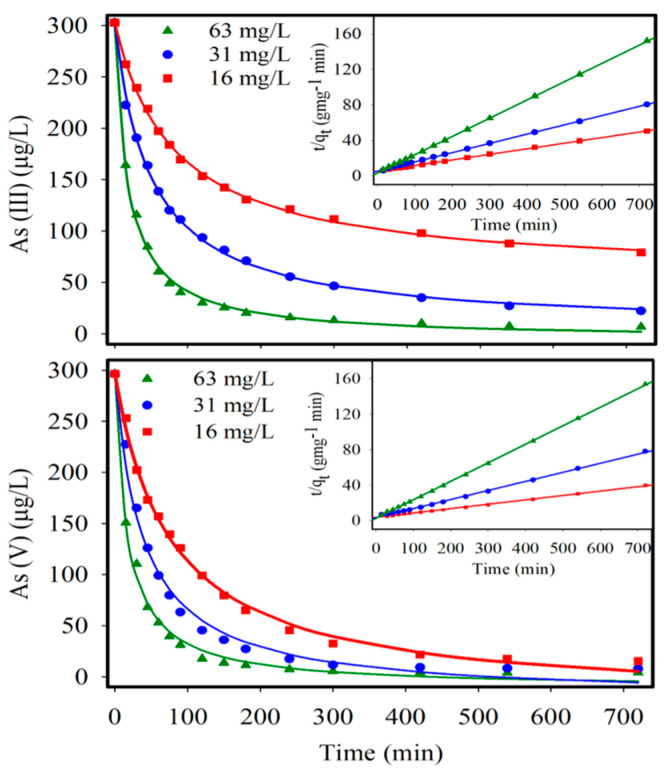
As(III) and As(V) adsorption kinetics with three different IONP loadings within CNF aerogel. The reported dosages are the IONP concentrations. The fitted aqueous arsenic concentrations were determined from the difference between the initial and adsorbed arsenic concentrations. The inset graphs are the pseudo second-order kinetic fits for the corresponding dosage of adsorbent according to Equation (6).

Figure 1 shows the adsorption kinetics of As(III) and As(V) for three CNF-IONP sorbent concentrations. Given that CNFs alone did not remove significant As(III) and As(V) concentrations, the reported sorbent dosages are those of IONPs. As(III) and As(V) concentrations decreased rapidly in the first 1–2 h depending on the sorbent dosage due to the increased number of available IONP surface sites. For both arsenic species, near-equilibrium adsorption was reached within ~3 h. IONP dosages of 63 mg L^−1^ resulted in the removal of As(III) and As(V) to <10 µg L^−1^ after 12 h of contact time.

The adsorption kinetic data were best represented by a pseudo second-order rate equation,

(6) $$\frac{t}{q_{t}}=\frac{t}{q_{e}}+\frac{1}{K_{\mathrm{ad}\;}q_{e}^{2}}\;$$

where *q*_e_ and *q*_t_ are the adsorbed arsenic (mg-As g-IONP^−1^) at equilibrium and at time *t*, respectively, and *K*_ad_ is the pseudo second-order rate constant (g-IONP mg-As.min^−1^). The *K*_ad_ values for As(III) and As(V) uptake increased with increasing adsorbent dosage (Table 1), indicating a faster uptake rate with a higher adsorbent concentration.

**Figure 2:**
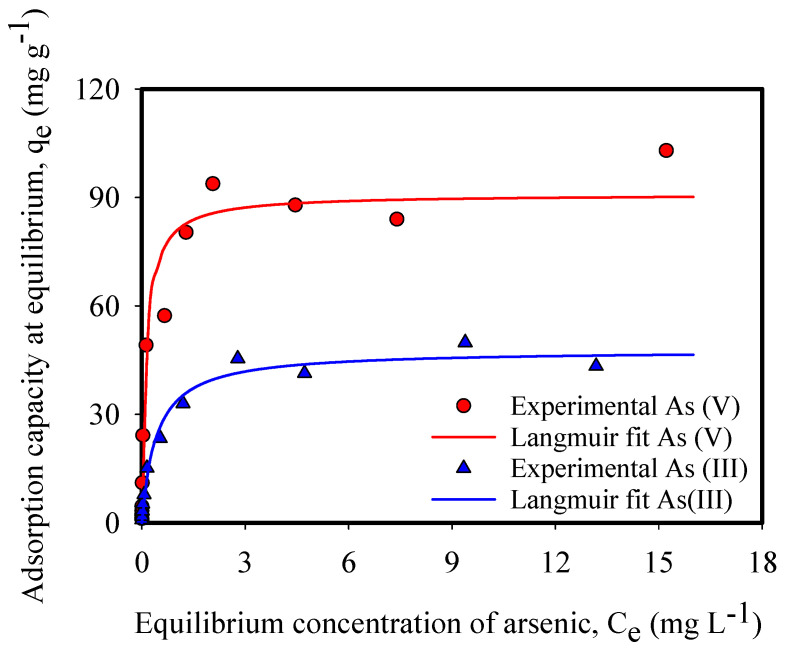
Equilibrium adsorption isotherms of As(III) and As(V). The adsorbent dosage was 63 mg of IONPs L^−1^ of arsenic ion.

The equilibrium adsorption of As(III) and As(V) onto CNF-IONP aerogels at pH 7 was investigated (Figure 2). Experimental data for both arsenic species were well represented by the Langmuir isotherm model (Equation (7)), which is applicable to a monolayer adsorption behavior assuming a uniform surface adsorption energy [23].

(7) $$q_{e}=\frac{q_{\max}K_{L}C_{e}}{1+K_{L}C_{e}}$$

where *q*_e_ is the mass of As(III) or As(V) adsorbed at equilibrium per mass of IONPs (mg-As g-IONP^−1^), *q*_max_ is the maximum adsorption capacity (mg-As g-IONP^−1^), *C*_e_ is the equilibrium As(III) or As(V) concentration (mg L^−1^) in post-adsorption water samples, and *K*_L_ is the Langmuir constant for adsorption. The maximum adsorption capacities of the CNF-IONP aerogel adsorbent were 47.75 and 90.90 mg-As g-IONP^−1^ for As(III) and As(V), respectively.

The constant *K_L_* in Equation (7) indicates the sorption affinity of a solute toward a sorbent. Our results show that As(V) has a significantly higher affinity toward the IONPs than As(III) (Table 2). The difference in affinity between the two arsenic species is especially evident at low aqueous arsenic concentrations that are representative of natural waters, indicating that As(III) is more mobile in the environment [24]. This may be attributed to the largely uncharged form of As(III) at pH 7 (H_3_AsO_3_ ⇔ H^+^ + H_2_AsO_3_^−^, p*K* = 9.15) compared to As(V) that is negatively charged at the same pH (H_3_AsO_4_ ⇔ H^+^ + H_2_AsO_4_^−^, p*K* = 2.3; H_2_AsO_4_^−^ ⇔ H^+^ + HAsO_4_^2−^, p*K* = 7.16). The negatively charged As(V) species interact more favorably with the positively charged surface of IONPs, resulting in a higher adsorption affinity and capacity (Table 2).

Findings from previous studies shown in Table 3 highlight the importance of functionalizing the micro- and nano-sized cellulosic materials to result in an acceptable affinity towards arsenic. The table also shows the superior performance of our adsorbent to other iron oxide-based nanoparticles used for arsenic removal. However, comparisons with iron oxide nanoparticles incorporated into cellulosic-based structures must be interpreted with caution. Of particular interest is the work of Yu et al. and Dong et al. [20,21], whose studies investigated iron oxide-based nanoparticles in cellulosic structures. The maximum adsorption capacity obtained herein is comparable to that of Yu et al. However, it is worth noting that the Fe_2_O_3_ content used in their study was 36%, which is approximately three times that of the IONP content used in this study (12.5%). The maximum adsorption capacity obtained by our adsorbent is considerably higher than that obtained by Dong et al. which could be attributed to the lower iron oxide content in their work (9.4%).

**Figure 3:**
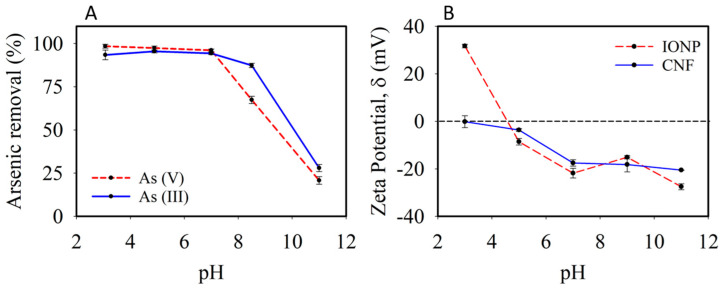
Effect of pH on arsenic removal percentage. Initial arsenic ion concentration: 1 mg L^−1^, IONP dosage: 63 mg of IONP L^−1^ (**A**) and zeta potential measurements of CNF and IONP suspensions at different pH values (**B**). Error bars represent standard deviation from triplet measurements.

The pH-dependent dissolved arsenic speciation affects its adsorption behavior. Figure 3A shows that a high removal rate (>93%) was achieved for both arsenic species within an acidic to neutral pH (3–7), but drastically dropped after pH 7. Given the circumneutral pH of drinking water, it can be expected that the CNF-IONP adsorbent can be used effectively to remove arsenic. Figure 3B shows the measured zeta potentials of CNFs and IONPs. The CNF surface is negatively charged within the pH range 3–11 due to the presence of carboxylate groups on the surface of CNFs [36]. The IONPs, however, have a low isoelectric point (IEP) of 4.6 compared to the 7–9 range reported for synthetic iron oxides [37]. This is due to the positive charge deficit brought about by the presence of divalent Mg^2+^ ions in the iron oxide structure [38]. The pH-dependent adsorption of arsenic species onto the IONPs may be explained by the opposite electrostatic charges between arsenic and the IONP surface at low pH values that favor adsorption [39] and electrostatic repulsion between the deprotonated surface groups of IONPs and the anionic arsenic species at higher pH values that result in diminished adsorption [40].

**Figure 4:**
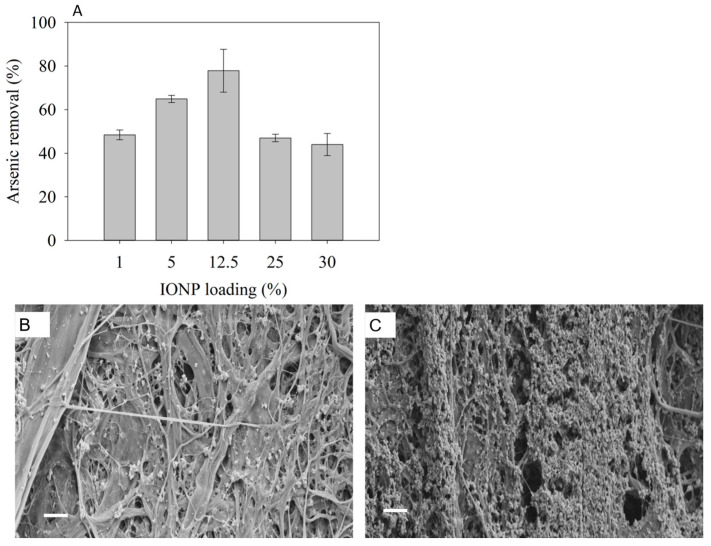
As(V) adsorption with different wt.% of IONPs in CNF-IONP aerogel. The error bars represent standard deviation from triplet measurements (**A**); SEM micrographs (scale bar: 1 µm) of 12.5 wt.% (**B**) and 25 wt.% (**C**) of IONPs in CNF-IONP aerogel.

The experiments to determine the effect of the IONP loading in the aerogel on adsorption were conducted using As(V). Figure 4A shows that the percent of arsenic removal was enhanced with the increase in IONPs up to 12.5 wt.%, above which, increasing the IONP concentration resulted in a decrease in arsenic removal. Note that the results presented earlier were obtained with 12.5 wt.% IONPs. A one-way ANOVA statistical test showed differences among the five groups of different IONP concentrations in CNF-IONP aerogel. Post hoc analysis indicated that the 12.5 wt.% sample exhibited maximum arsenic removal. The decrease in arsenic removal with an increase in IONP loading suggests that higher IONP loadings may result in densely packed IONPs in the aerogel, resulting in a decreased surface area for adsorption. High-resolution SEM images of CNF-IONP aerogels at 12.5 and 25 wt.% IONP loadings (Figure 4B,C) show that an increasing IONP concentration can lead to particle aggregation at the surface. A similar observation was reported in a study where increasing the magnetite concentration beyond a threshold value decreased Cr(VI) adsorption, and the observation was attributed to the aggregation of nanoparticles and the reduction in the availability of active adsorption sites [41].

The stability of IONPs in the aerogel was investigated through the measurement of leached iron upon soaking the aerogels in water under constant agitation. For the 12.5 wt.% IONPs in the CNF-IONP aerogel, an insignificant amount (<10 µg L^−1^) of iron was leached at pH 7, which is below the WHO and US-EPA MCL of 300 µg L^−1^ for iron concentration in drinking water.

### 3.2. Characterization of the Adsorbent

**Figure 5:**
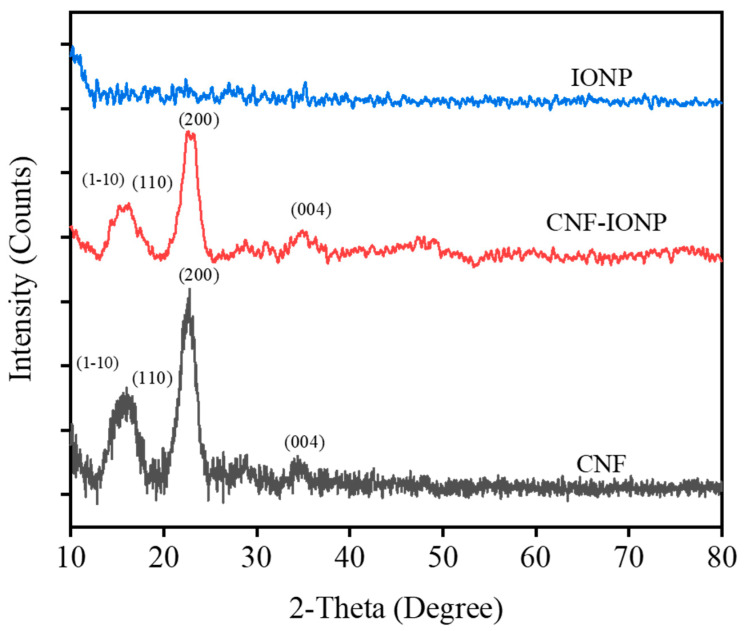
XRD spectra of the freeze–dried IONP, CNF–IONP, and CNF aerogels.

Figure 5 shows the XRD patterns for freeze-dried IONP, CNF-IONP, and CNF aerogels. The XRD pattern of freeze-dried IONPs showed no peaks, indicating the amorphous nature of the nanoparticles. The XRD peaks of both CNF and CNF-IONP aerogels were similar. The broad peak between 15° and 16.5° corresponds to Miller indices (1–10) and (110), respectively, and the sharp peak around 22.5° corresponds to the lattice diffraction of the (200) plane [42]. The low-intensity peak around 35° corresponds to the plane (004) of cellulose I [43]. A noticeable decrease was observed in peak intensities for cellulose in CNF-IONP aerogel resulting from the incorporation of the IONPs.

**Figure 6:**
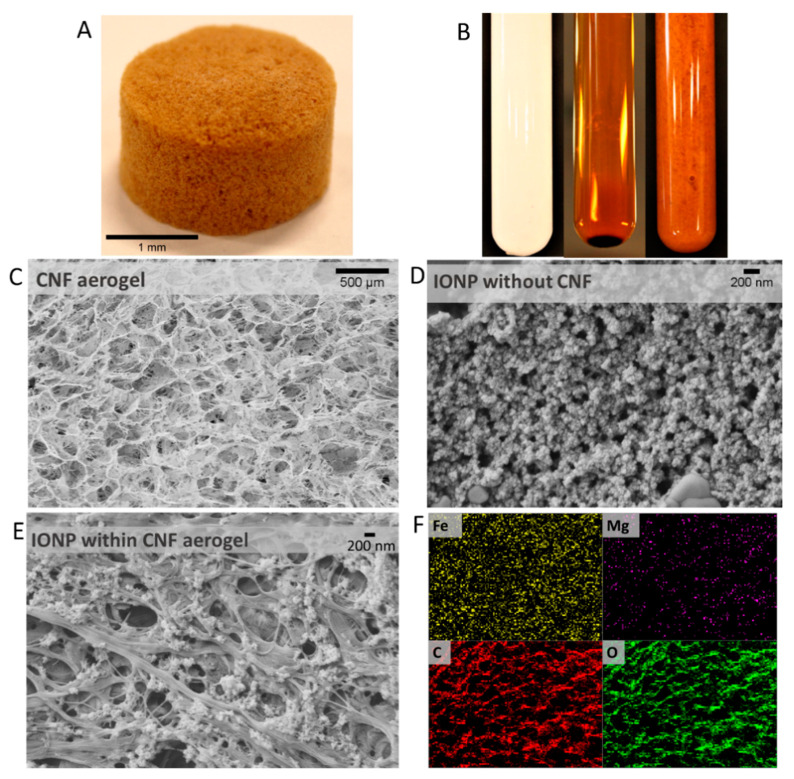
Photograph of CNF-IONP aerogel (**A**); from left to right: CNF suspension of 1 wt.% solids content, aggregated and precipitated IONP suspension, and IONPs immobilized on CNFs (**B**); SEM image of CNF aerogel macrostructure without IONP (**C**); SEM image of IONP (**D**); SEM image of 12.5% CNF-IONP aerogel (**E**); Corresponding EDS elemental mapping for CNF-IONP (scale bar: same as (**C**)) (**F**). Note the different scales.

Additional SEM analysis was conducted to evaluate the internal structure and morphology of IONP, CNF, and CNF-IONP aerogels (Figure 6). Figure 6A shows a photograph of the CNF-IONP aerogel. The IONP suspension precipitated in the absence of CNFs due to their tendency to aggregate (Figure 6B). However, in the presence of CNFs, the IONPs did not precipitate. Instead, the IONPs were immobilized on the cellulose fibrils (Figure 6B). Without IONPs, CNFs form a highly porous structure resulting from freeze-drying of CNF suspensions (Figure 6C). The role of CNFs in immobilizing the IONPs on their surfaces was further verified by observing the IONPs in the presence and absence of CNFs using SEM. Figure 6D shows the freeze-dried IONP aggregates. The stable CNF suspension immobilized the IONPs on the fibrils, resulting in uniformly distributed nanoparticles within the aerogel (Figure 6E). This finding was further supported by EDS analysis (Figure 6F) of the aerogel, which verified the uniform distribution of the IONPs.

The porosity values of CNF and CNF-IONP aerogels were 98% and 95%, and their densities were 0.009 ± 0.01 and 0.013 ± 0.03 g cm^−^^3^, respectively. The water absorption capacities for the CNF and CNF-IONP aerogels were 96.5 ± 4.9 and 74.9 ± 2.6 g g^−1^, respectively. These results are in agreement with previously reported CNF aerogels of similar densities [44].

The freeze-dried IONPs exhibited a considerably high specific surface area of 165 m^2^ g^−1^. The BET surface area of CNF aerogel without additives is 13 m^2^ g^−1^, which is typical for this type of material [45]. The minimal aggregation and uniform distribution of the IONPs in the aerogel suggest that the surface area of the IONPs was not compromised. The surface area of the amorphous IONPs in this study is also higher than those of crystalline iron oxides such as hematite and maghemite [4,6] and comparable to other doped iron oxide nanoparticle adsorbents [4].

**Figure 7:**
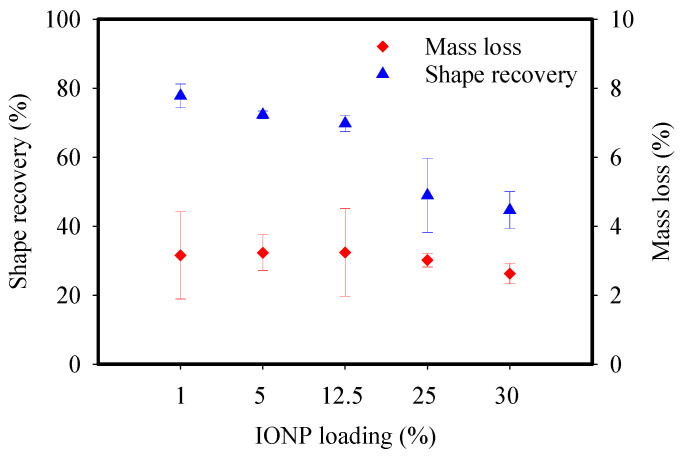
Shape recovery and mass loss of CNF-IONP aerogels at various IONP loadings. Error bars represent standard deviation from triplet measurements.

The structural stability of CNF-IONP aerogels when soaked in water and under constant agitation was assessed through mass loss measurements. The results showed that the <3% mass loss of aerogels was not statistically different (*p* > 0.05) under various IONP loadings (Figure 7). This result shows that regardless of the loading of IONPs, they maintain their contact with the fibrils, which renders the aerogel stable even with constant agitation for 12 h. We further supported this result by assessing the leaching of iron under the same conditions. The iron concentration in water after soaking the aerogels increased by <10 μg L^−1^ at pH 7. Although the leaching of iron is minimal, this result revealed the importance of rinsing the aerogels with water prior to their intended application.

In addition, the aerogels exhibited an interesting shape recovery pattern resulting from the addition of the epichlorohydrin crosslinker. The key role of the crosslinker is to impart adequate stability upon soaking the aerogels in water. Typically, CNF-based aerogels without a crosslinker tend to disintegrate in water. Here, when the CNF-IONP dry aerogel is compressed into a thin disk, at least 50% of the shape is recovered upon soaking in water. The shape recovery percentage ranged from 50% to 80% depending on the quantity of IONPs (Figure 7); a higher loading of IONPs decreased the shape recovery. Typically, in crosslinked CNF-only aerogels, this phenomenon is attributed to the high surface tension of water, which creates large capillary forces that expand the fibrils. Additionally, the diffusion of water into the pores coupled with partial swelling of amorphous cellulose augment the extent of recovery [44,45,46]. It is not surprising that the presence of the IONPs decreased the shape recovery percentage, which is presumably due to the partial disruption of the CNF network, which renders the recovery more challenging.

**Figure 8:**
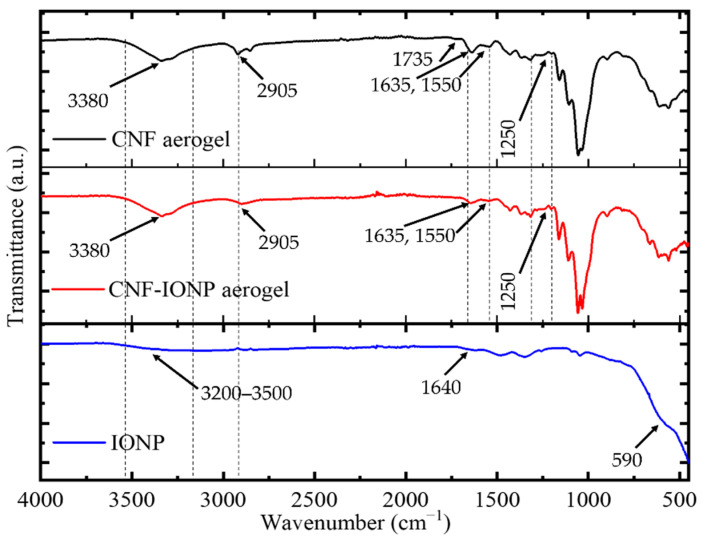
FTIR spectra for freeze-dried IONP, crosslinked CNF, and CNF-IONP aerogels.

To investigate the interaction between CNFs and IONPs, FTIR spectroscopy was performed (Figure 8). In freeze-dried IONP sample, the peak at ~590 cm^−1^ is attributed to Fe–O band vibration [47,48]. The broad peaks at 3200–3500 and ~1640 cm^−1^ were assigned to the stretching and bending vibrations of the OH group, respectively [49]. On the other hand, CNF and CNF-IONP aerogels exhibited almost similar IR spectra. The broad peak around 3380 cm^−1^ corresponds to OH stretching of CNFs [50]. The N–H stretching, which originated from the crosslinking reaction [51], overlaps with the OH stretching peak. Figure S2 shows the structure of the crosslinker and the proposed crosslinking reaction. The peak at 2905 cm^−1^ is attributed to the aliphatic C–H stretching [52]. The peaks at 1635 and 1550 cm^−1^ correspond to amide I and II, respectively [53]. In addition, the broad peak around 1250 cm^−1^ may be assigned to C–O stretching vibration for ester bonds due to the crosslinking [53]. The intensity of this peak is lower in non-crosslinked CNF samples compared to the crosslinked samples (Figure S3).

Although the CNF-IONP aerogels showed excellent stability in water aided by the crosslinker, no characteristic peak at ~1735 cm^−1^, which is attributed to C=O stretching vibration for the ester group [53], was observed. However, this peak was visible in the crosslinked CNF aerogels, which can be disrupted by the presence of IONPs. The FTIR spectra of the CNF aerogels before and after crosslinking are presented in Figure S3. Besides this, no noticeable change was observed in the FTIR spectra of CNF-IONP aerogel compared to CNF aerogel, which indicates that there is no evidence of chemical reaction between CNFs and IONPs. It is somewhat surprising that despite the absence of evidence for a chemical reaction, IONPs are retained within the CNF structure remarkably well. This suggests that, in addition to Van der Waals interactions between CNFs and IONPs, other factors may be contributing to the stability of IONPs within the aerogel such as mechanical interlocking [54]. It also suggests that the attraction forces between CNFs and IONPs are stronger than those between the IONPs.

## 4. Conclusions

A novel and highly efficient CNF-IONP aerogel was prepared by incorporating IONPs into CNFs by simultaneous freeze-drying. This method addresses the previous issues of nanoparticle aggregation to enable the application of nanoparticle adsorbents in fixed-bed columns at the industrial scale. The amorphous nature of the IONPs was verified by means of XRD analysis. The IONPs had a remarkably high specific surface area (165 m^2^ g^−1^). The 12.5 wt.% IONPs in the CNF aerogel resulted in a maximum arsenic removal and uniform distribution on the cellulose fibrils. The maximum adsorption capacities of CNF-IONP aerogel for As(III) and As(V) were 48 and 91 mg As g-IONP^−^^1^, respectively. The adsorbent was stable in water and under constant agitation with negligible mass loss. In addition, the adsorbent exhibited a partial shape recovery functionality that could be advantageous for transportation purposes. Overall, this study will also provide a near-term alternative to efficient and bio-based commercial adsorbents for arsenic removal from water.

## Figures and Tables

**Table 1:** Summary of kinetic parameters for As(III) and As(V) adsorption on IONPs in CNF-IONP aerogel.

Adsorption Parameters	As(III)			As(V)		
Initial As Concentration (mg-As L^−1^)	0.0296			0.0302		
Adsorbent Loading (mg-IONP L-As^−1^)	16	31	63	16	31	63
*q*_e_ (mg-As g-IONP^−1^)	15.87	9.57	4.85	20.41	9.78	4.78
*K*_ad_ (g-IONP mg-As·min^−1^)	7.6 × 10^−4^	2.1 × 10^−3^	1.35 × 10^−2^	6.7 × 10^−4^	3.2 × 10^−3^	1.67 × 10^−2^
*r* ^2^	0.99	0.99	0.99	0.99	0.99	0.99

**Table 2:** Summary of Langmuir isotherm model fits for arsenic adsorption onto the CNF-IONP aerogel.

	As(III)	As(V)
*q_max_* (mg-As g-IONP^−^^1^)	47.75	90.90
*K_L_*	2.36	7.95
*r* ^2^	0.99	0.99

**Table 3:** Arsenic adsorption capacities of cellulose- and iron oxide-based adsorbents from the literature.

Adsorbent	As Concentration Range, *C*_0_ (mg L^−1^)	Maximum as Adsorption Capacity, *q_max_* (mg g^−1^)	BET Surface Area (m^2^ g^−1^)	pH	Reference
Cellulose-g-PDMAEMA	As(III) & As(V): 0.05–8.9	As(III): 8.96; As(V): 27.93	—	<10	[25]
FeOOH/CuO@WBC	As(III): 20–200	As(III): 76.1	—	3.5	[26]
DETA-g-DA-NCC	As(III) & As(V): 0.005–50	As(III): 10.56; As(V): 12.06	—	7.5	[27]
Functionalized CNFs	As(V): 0.025–40	As(V): 24.9	0.16	4–8	[28]
Cellulose-g-GMA-b-TEPA	As(III): ~5–35; As(V): ~20–100	As(III): 5.71; As(V): 75.13	3.68	As(III): 7 & As(V): 5	[29]
Fe(III)-AM-PGMACell	As(V): 25–400	As(V): 78.8	39.9	6	[30]
AM-Fe-PGDC	As(V): 10–400	As(V): 105.47	31.6	6	[31]
Cell-N-Cu	As(V): 100–700	As(V): 98.9	—	8.4	[32]
CNs/Fe_2_O_3_ nanorod	—	As(III): 13.87, As(V): 15.71	—	7 & 3	[21]
Cellulose@Fe_2_O_3_ composites *	—	As(III): 64.33, As(V): 89.19	113	7	[20]
iMNP	As(V): 1–10	As(V): 12.74	145.5	6.6	[33]
Fe_3_O_4_/AC composite	As(III): 2–120	As(III): 7.5	—	8	[34]
OMIM	As(III) & As(V): 1–100	As(III): 67.89, As(V): 93.54	154	3	[35]
IONP@CNF-IONP aerogel *	As(III): 0.055–15.9; As(V): 0.073–21.7	As(III): 47.75; As(V): 90.90	165	7	This work

Abbreviations: Cellulose-g-PDMAEMA: native cellulose fibers modified with poly(N,N-dimethyl aminoethyl methacrylate), FeOOH/CuO@WBC: iron(III) oxyhydroxide/copper oxide composite based on water bamboo cellulose, DETA-g-DA-NCC: diethylene triamine grafted dialdehyde nanocrystalline cellulose, Functionalized CNFs: cellulose nanofibrils modified with trimethylammonium chloride, Cellulose-g-GMA-b-TEPA: glycidyl methacrylate grafted cellulose modified with tetraethylenepentamine, Fe(III)-AM-PGMACell: iron(III)-coordinated amino-functionalized poly(glycidyl methacrylate)-grafted cellulose, AM-Fe-PGDC: Fe(III)-coordinated amino-functionalized poly(glycidylmethacrylate)-grafted TiO_2_-densified cellulose, Cell-N-Cu: copper containing modified cellulose, CNs/Fe_2_O_3_ nanorod: cellulose nanocrystals/iron oxide nanorod composite, Cellulose@Fe_2_O_3_ composites: composite material containing magnetic Fe_2_O_3_ and cellulose iMNP: magnetic nanoparticles prepared from iron containing sludge, Fe_3_O_4_/AC composite_3_: iron oxide/commercial activated carbon composite, OMIM: mesoporous iron manganese bimetal oxides. * The maximum adsorption capacities are based on the iron oxide only.

## Supplementary Materials

The following are available online at https://www.mdpi.com/article/10.3390/nano11112818/s1, Figure S1: The summary of preparatory scheme for CNF-IONP aerogel; Table S1: Summary of aerogel formulation before and after the incorporation of IONPs; Figure S2: Proposed reaction mechanism between cellulose and Polycup^TM^; Figure S3: The FTIR spectrum of crosslinked and non-crosslinked CNF aerogel.
