# Supplementary material for: Highly Efficient Iron Oxide Nanoparticles Immobilized on Cellulose Nanofibril Aerogels for Arsenic Removal from Water
Source: Nanomaterials (Basel). 2021 Oct 23;11(11):2818. doi: 10.3390/nano11112818 (PMC8623684; doi:10.3390/nano11112818)
Supplement: Supplementary file 1 [file nanomaterials-11-02818-s001.zip › nanomaterials-1431769-supplementary.pdf]

nanomaterials

MDPI

Supplementary Materials

Highly Efficient Iron Oxide Nanoparticles Immobilized on Cellulose Nanofibril Aerogels for Arsenic Removal from Water

Md Musfiqur Rahman [1], Islam Hafez [1,*], Mehdi Tajvidi [1] and Aria Amirbahman [2]

[1] Laboratory of Renewable Nanomaterials, School of Forest Resources, University of Maine, 5755 Nutting Hall, Orono, ME 04469, USA; md.musfiqur.rahman@maine.edu (M.M.R.); mehdi.tajvidi@maine.edu (M.T.)
[2] Department of Civil, Environmental and Sustainable Engineering, Santa Clara University, 500 El Camino Real, Santa Clara, CA 95053, USA; aamirbahman@scu.edu
* Correspondence: islam.hafez@maine.edu (I.H.)

Table S1. Summary of aerogel formulation before and after the incorporation of IONPs.

	CNF		IONPs		Crosslinker (CL)		Adsorbent
**Sample Name**	**CNF wt. %**	**CNF Dry Mass (mg)**	**IONP wt. %**	**IONP Dry Mass (mg)**	**CL wt. % (Based on Dry CNF)**	**CL Dry Mass (mg)**	**Adsorbent Dry Mass (mg)**
CNF Aerogel	100	35	0	5	5	0.07	35.07
CNF-IONP Aerogel	87.5	35	12.5	5	5	0.07	40.07

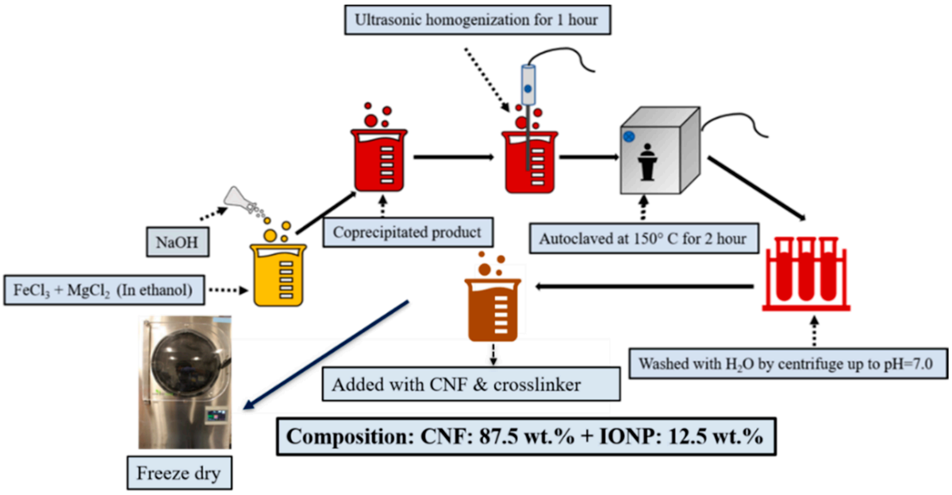

Figure S1. The summary of the preparatory scheme for CNF-IONP aerogel.

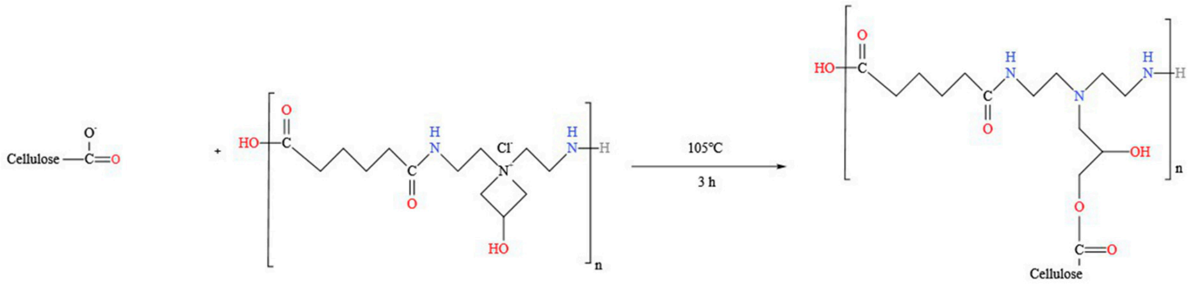

Cellulose with carboxylate group | Polycup™ (polyamide-epichlorohydrin) 5150 crosslinker | Cellulose crosslinked with Polycup™ (polyamide-epichlorohydrin) 5150 crosslinker

Figure S2. Proposed reaction mechanism between cellulose and Polycup™.

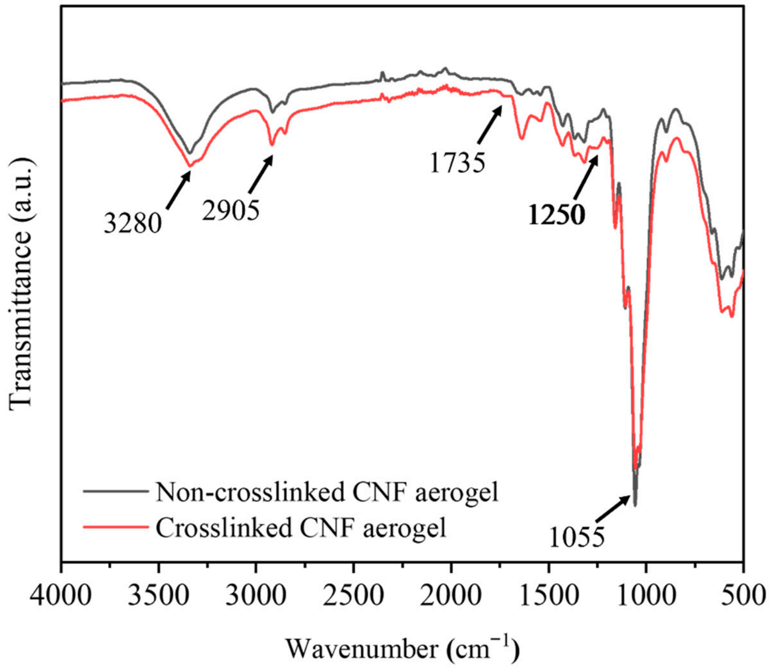

Figure S3. The FT-IR spectrum of crosslinked and non-crosslinked CNF aerogel.
